# The Effect of *Lactobacillus gasseri* BNR17 on Postmenopausal Symptoms in Ovariectomized Rats

**DOI:** 10.4014/jmb.2105.05032

**Published:** 2021-07-19

**Authors:** Sol Lee, Dong Hoon Jung, Miri Park, Seung-Woo Yeon, Sang-Hyuk Jung, Sung-Il Yun, Han-Oh Park, Wonbeak Yoo

**Affiliations:** 1 AceBiome Inc., Seoul 06164, Republic of Korea; 2 R&D Center, AceBiome Inc., Daejeon 34013, Republic of Korea; 3 siRNAgen Therapeutics, Daejeon 34302, Republic of Korea; 4 Bioneer Corporation, Daejeon 34302, Republic of Korea

**Keywords:** *Lactobacillus gasseri* BNR17, menopause symptoms, ovariectomized rats

## Abstract

Clinical and preclinical studies have reported that *Lactobacillus gasseri* BNR17, a probiotic bacterial strain isolated from human breast milk, reduces body weight and white adipose tissue volume. In order to further explore the actions of *L. gasseri* BNR17, we investigated the anti-menopausal effects of *L. gasseri* BNR17 in an ovariectomized (OVX) rat model. The serum alanine aminotransferase levels of the rats in the OVX-BNR17 group were lower than those of the rats in the OVX-vehicle only (OVX-Veh) group. Upon administration of *L. gasseri* BNR17 after ovariectomy, calcitonin and Serotonin 2A levels increased significantly, whereas serum osteocalcin levels showed a decreasing tendency. Compared to the rats in the OVX-Veh group, those in the OVX-BNR17 group showed lower urine deoxypyridinoline levels, lower pain sensitivity, and improved vaginal cornification. Furthermore, *L. gasseri* BNR17 administration increased bone mineral density in the rats with OVX-induced femoral bone loss. These results suggest that *L. gasseri* BNR17 administration could alleviate menopausal symptoms, indicating that this bacterium could be a good functional probiotic for managing the health of older women.

## Introduction

Menopause is a natural process in older women in whom the activities of estrogen and progesterone secreted from the ovaries has decreased. Menopause causes physical and psychological changes that result in osteoporosis, pain sensitivity, mood disorder, vaginal dryness, and cardiovascular disease [1, 2]. As the life expectancy of women has increased, most women typically spend more than one-third of their lives experiencing postmenopausal symptoms [3]. The population of menopausal women has been predicted to increase to 1.2 billion by 2030, with the number increasing by 47 million each year [4]. Although hormone replacement therapies (HRTs) involving calcitonin and estrogen are well-known methods to improve menopausal symptoms, long-term use of HRT has some risks, such as breakthrough bleeding, breast tenderness, bloating, nausea, and increased risk of breast and endometrial cancer [5-8]. Thus, effective prevention or treatment options for menopausal symptoms are needed.

In the recent years, there has been a rapid increase in studies investigating the usage of probiotics to relieve menopausal symptoms without noticeable side effects [9]. Probiotics are live microorganisms that provide various health benefits and are considered functional foods when administered in adequate amounts [10-13]. Recently, consumption of probiotics has been shown to improve menopause-related symptoms, such as bone health disorders, stress-induced sleep disturbance, and depression disorder [14-17]. Despite the increasing evidence, probiotics are considered helpful in preventing and improving menopausal symptoms.

*Lactobacillus gasseri* BNR17 was originally isolated from human breast milk and has been reported to play a functional role in irritable bowel syndrome, weight control, and type 2 diabetes [18-21]. In the present study, we aimed to discover the role of *L. gasseri* BNR17 in menopause in an ovariectomized (OVX) rat model. This study demonstrated that *L. gasseri* BNR17 administration improves various symptoms of menopause and also increases bone mass in OVX rats.

## Materials and Methods

### Materials

*Sophora japonica* fruit extracts (SJFE, containing 100–150 mg/g of sophoricoside) was purchased from BTC Co.(Korea) and was used in creating the positive control for menopausal symptoms reduction [22, 23]. *L. gasseri* BNR17 (Accession No: KCTC10902 BP) was prepared according to previous reports [24] with slight modifications, such as using general cytoprotectants for freeze-drying of lactic acid bacteria. Centrifuged *L. gasseri* BNR17 pellets were mixed with cryoprotectants containing skim milk, trehalose, dextrin, and fructo-oligosaccharide and then freeze-dried. The dried pellets were stored at -70 °C until further use. All other chemicals were of the highest analytical grade available.

### Animals and Experiments

Nine-week-old sham-operated (Con, *n* = 6) or ovariectomized (OVX, *n* = 24) female Sprague-Dawley (SD) rats were purchased from Daehan Biolink Co., Ltd. (Korea). The mice were kept under controlled environmental conditions (23 ± 12°C, 55 ± 5% humidity, 12-h light/dark cycle) with free access to food and water at the Animal Care Facility at siRNAgen Therapeutics Corporation (Korea). After a one-week acclimation period, Con received a standard diet (2918C, Harlan Teklad, USA), while OVX received a high sucrose diet (HSD, AIN-76A Purified Diet, USA). HSD-fed OVX rats were randomly assigned to three groups as follows: (1) vehicle only (Veh, *n* = 8); (2) *L. gasseri* BNR17 at 1 × 10^10^ CFU/rat (BNR17, *n* = 8); (3) SJFE at 100 mg/kg/rat (SJFE, *n* = 8) and were orally administered suspensions of 100 μl dissolved in phosphate-buffered saline (PBS) twice per day. The treatment doses were determined based on previous studies [23, 24]. After 14 weeks of administration, the rats were sacrificed, and their blood was collected for further biochemical analysis. Urine samples were collected at the beginning and the end of the experimental period. The microarchitecture of the femur was analyzed using a Quantum GX μ-CT imaging system (PerkinElmer, USA) at the Korea Basic Science Institute (KBSI, Korea). Vaginal smears were collected from all rats. The smear samples were stained with methylene blue for observation and analysis as described previously [25]. Mechanical sensitivity was measured using von Frey filaments (JD-SI-11F, Jeung do bio & Plant Co. Ltd., Korea) on the mesh-bottom cages in the left hind paw, using an up-and-down method [26]. The protocol used was approved by the Committee on the Ethics of Animal Experiments of the siRNAgen Therapeutics Corporation (AEC-20200319-000). The siRNAgen therapeutics-IACUC was approved by the Animal and Plant Quarantine Agency (Korea).

### Biochemical Analyses

Clinical chemistry parameters, including alanine aminotransferase (ALT), aspartate aminotransferase (AST), blood urea nitrogen (BUN), creatinine (Crea), and albumin (ALB), were measured using automatic analyzers (BS-220, Mindray, China; ADVIA 2120i, Siemens, Germany) at KP&T (Cheongju-si, Chung-cheong bukdo, Korea). Metabolic parameters including total cholesterol (T-Chol), triglyceride (TG), glucose (GLU), and low-density lipoprotein cholesterol (LDL) were also determined at KP&T as described above. The concentrations of estradiol (E2, CSB-E05110r), osteocalcin (CSB-E05129r), calcitonin (CSB-E05132r), estradiol (CSB-E05110r), and Serotonin 2A (5-HT-2A, CSB-E14956r) were assessed using an ELISA kit (CUSABIO, China). The level of urinary deoxypyridinoline (DPD) was measured using a rat deoxypyridinoline (DPD) ELISA Kit (CSB-E08400r, CUSABIO, China). All experiments were performed according to the manufacturer’s protocols, and the results were analyzed using a microplate reader (Infinite M200 PRO, TECAN, Switzerland).

### Statistical Analysis

The data were expressed as mean values with their standard errors. Analyses were performed using the Student’s *t*-test or the one-way analysis of variance test for multiple comparisons. For all comparisons, *p < 0.05 and **p < 0.01 were considered statistically significant.

## Results

### Effect of *L. gasseri* BNR17 Administration on Biochemical Parameters in Serum and Urine

**Fig. 1:**
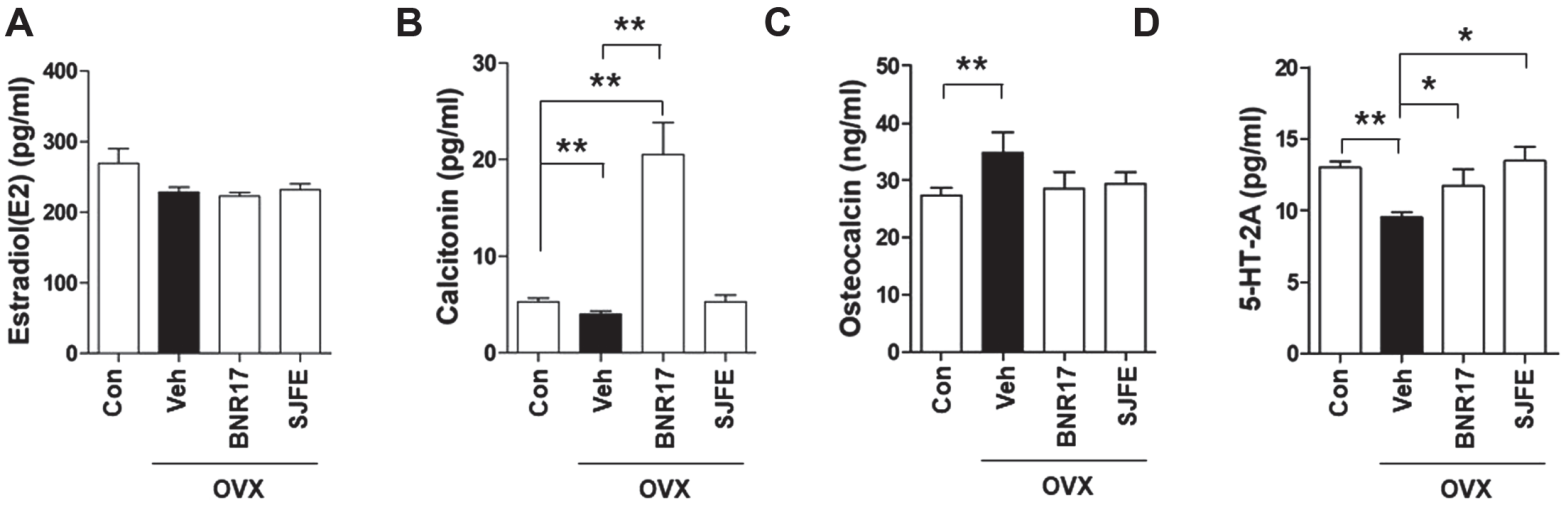
Effects of *L. gasseri* BNR17 administration on serum levels of menopausal hormones in OVX rats. Serum estradiol (E2) (**A**), Calcitonin (**B**), Osteocalcin (**C**), and 5-HT-2A (**D**) were measured after 14 weeks of initial administration (*n* = 6~8 rats per group). *Sophora japonica* fruit extract (SJFE) treatment was used to create the positive control.

**Fig. 2:**
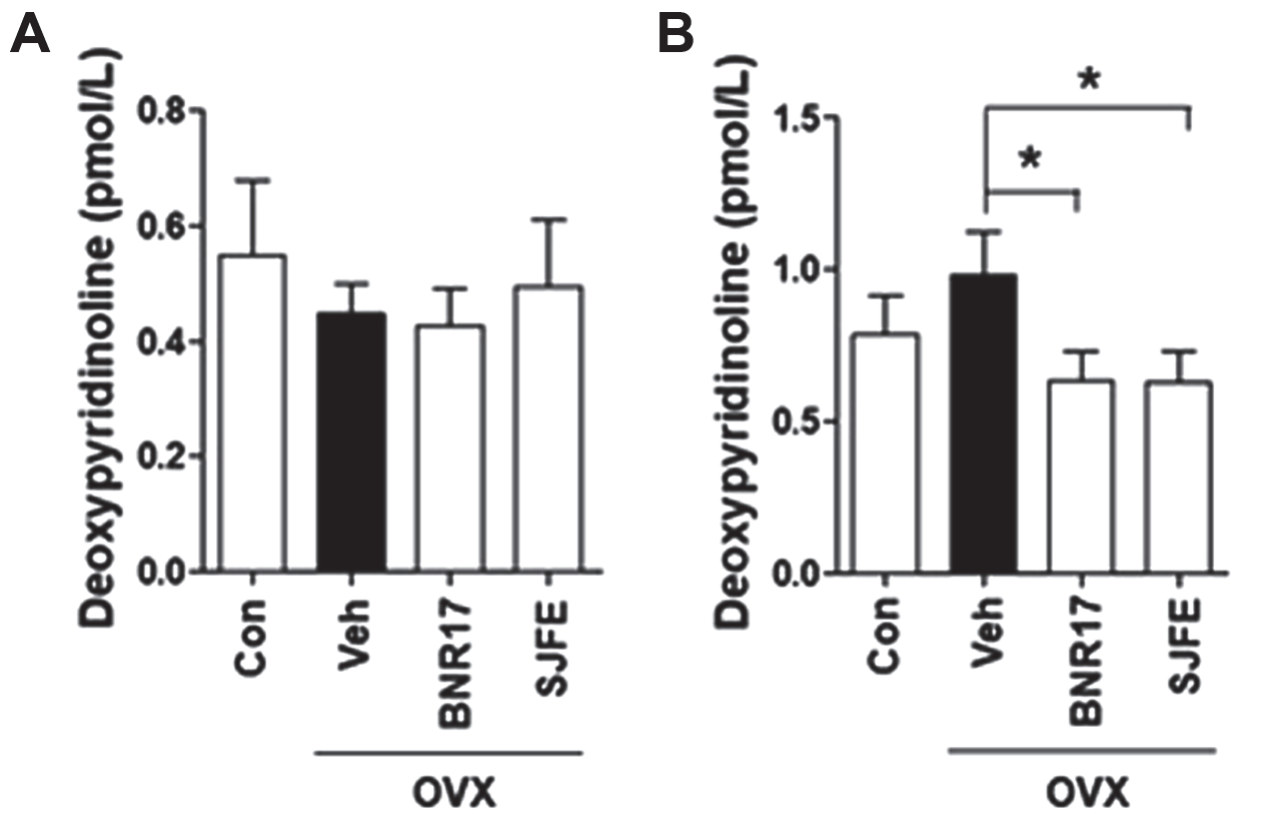
Effects of 14-week administration of *L. gasseri* BNR17 on urine deoxypyridinoline levels in OVX rats. The urine concentration of deoxypyridinoline before (**A**) and after (**B**) the experimental period (*n* = 6~8 rats per group). SJFE treatment was used to create the positive control.

To confirm the establishment of the OVX model in rats, the concentrations of estradiol (E2) in the serum were measured. As shown in Fig. 1A, the OVX group showed lower serum levels of estradiol (E2) than the control group. Previous studies have shown that the level of ALT, a liver injury marker, was increased in OVX models [27]; therefore, in the present study, further serum biochemical analysis was performed. Administration of *L. gasseri* BNR17 significantly reduced the serum levels of ALT and Cre when compared to those in the Veh-treated group (Table 1), although the weight of organs, including the liver, lung, heart, kidney, and the spleen, did not differ among groups (data not shown). Next, the effect of *L. gasseri* BNR17 on serum markers of bone turnover was investigated. Treatment with BNR17 significantly increased serum calcitonin levels (Fig. 1B); however, the levels of osteocalcin tended to decrease (*p* = 0.06) after treatment with *L. gasseri* BNR17 (Fig. 1C). As 5-HT-2A plays an important role in the state of depressive disorder in perimenopausal and postmenopausal women [28-30], we performed further biochemical analysis, investigating serum 5-HA-2A levels. Interestingly, 5-HT-2A levels were attenuated following *L. gasseri* BNR17 treatment (Fig. 1D). Subsequently, we investigated the effect of *L. gasseri* BNR17 administration on urine deoxypyridinoline. Urine deoxypyridinoline is a well-established marker of collagen degradation and bone resorption [31, 32]. No change in urinary deoxypyridinoline levels was observed in all groups at the beginning of the experimental period (Fig. 2A), whereas *L. gasseri* BNR17 administration effectively lowered the concentration of urine deoxypyridinoline when compared to that in the Veh-treated group at the end of the experimental period (Fig. 2B). These findings demonstrate that *L. gasseri* BNR17 treatment attenuates bone turnover and improves hepatic function and depression-like symptoms in OVX rat models.

### Effect of *L. gasseri* BNR17 Administration on Mechanical Response Thresholds and Vaginal Cornification

**Fig. 3:**
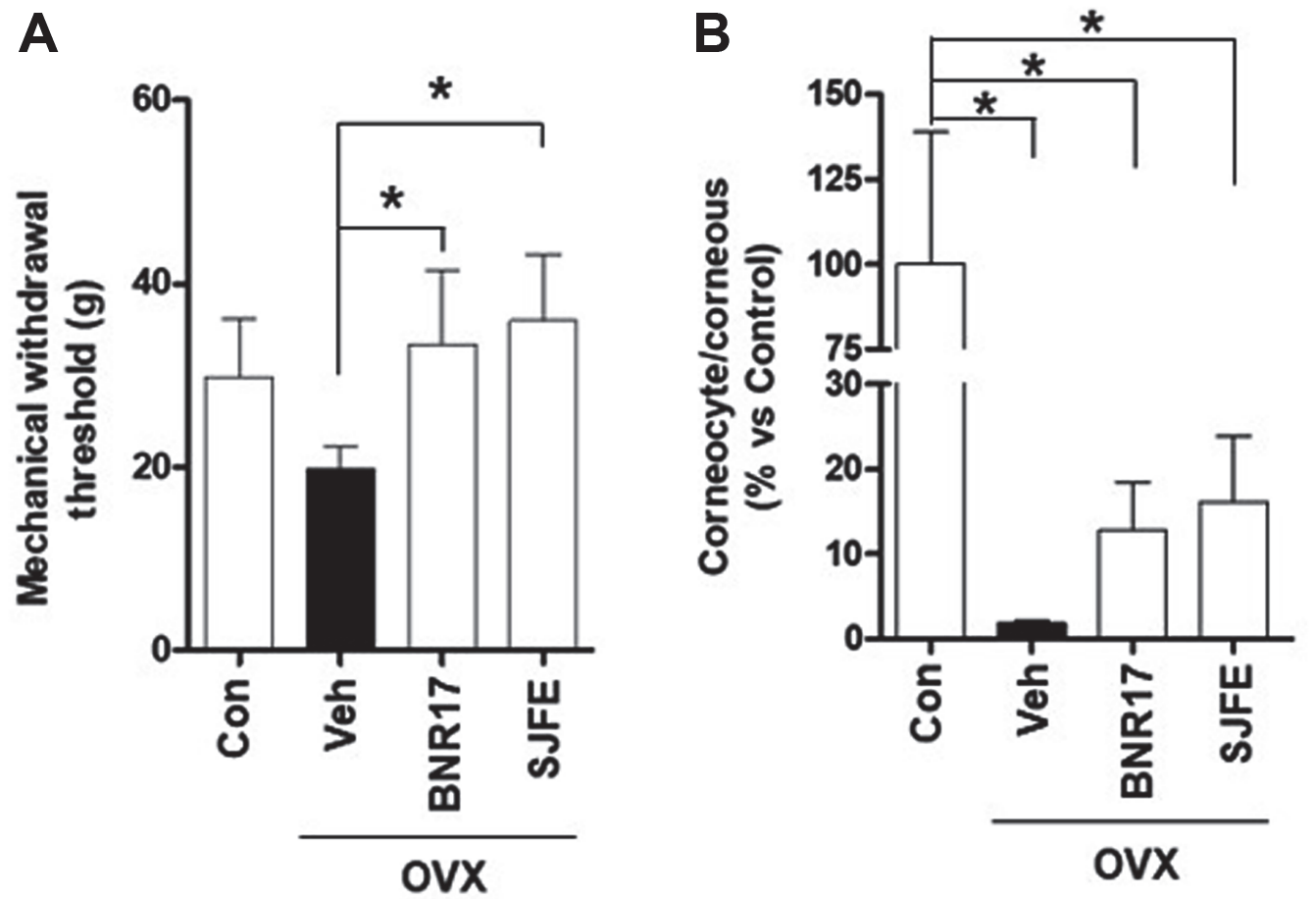
Mechanical threshold of paw withdrawal and the vaginal smear test. (**A**) The von Frey tests were carried out 14 weeks after *L. gasseri* BNR17 administration. (**B**) Vaginal smears were methylene blue-stained and measurements were obtained percent of corneocyte/corneous (*n* = 6~8 rats per group). SJFE treatment was used to create the positive control.

OVX animals, which are widely and well-established osteoporosis animal models, show less vaginal cornification and hyperalgesia to mechanical stimuli [25, 26, 33-36]. Therefore, we conducted pain-related behavioral tests in the four experimental groups. The von Frey test performed at the end of the experiment showed that the Veh-administered group had the lowest withdrawal threshold to mechanical stimuli among all the groups. The withdrawal threshold of the BNR17 treated group was significantly higher than that of the Veh-treated group, which indicated that *L. gasseri* BNR17 significantly alleviated pain-like behavior in OVX rats (Fig. 3A). To test whether *L. gasseri* BNR17 affects vaginal cornification, we investigated the distribution of cornified cells. As shown in Fig. 3B, the mean percentage of vaginal cornification observed in the BNR17 group was higher than that in the Veh group (*p* = 0.06). As a result, we speculate that treatment with *L. gasseri* BNR17 can relieve OVX-induced menopausal symptoms, including pain sensitivity and vaginal cornification.

### Effect of *L. gasseri* BNR17 Administration on Bone Density

**Fig. 4:**
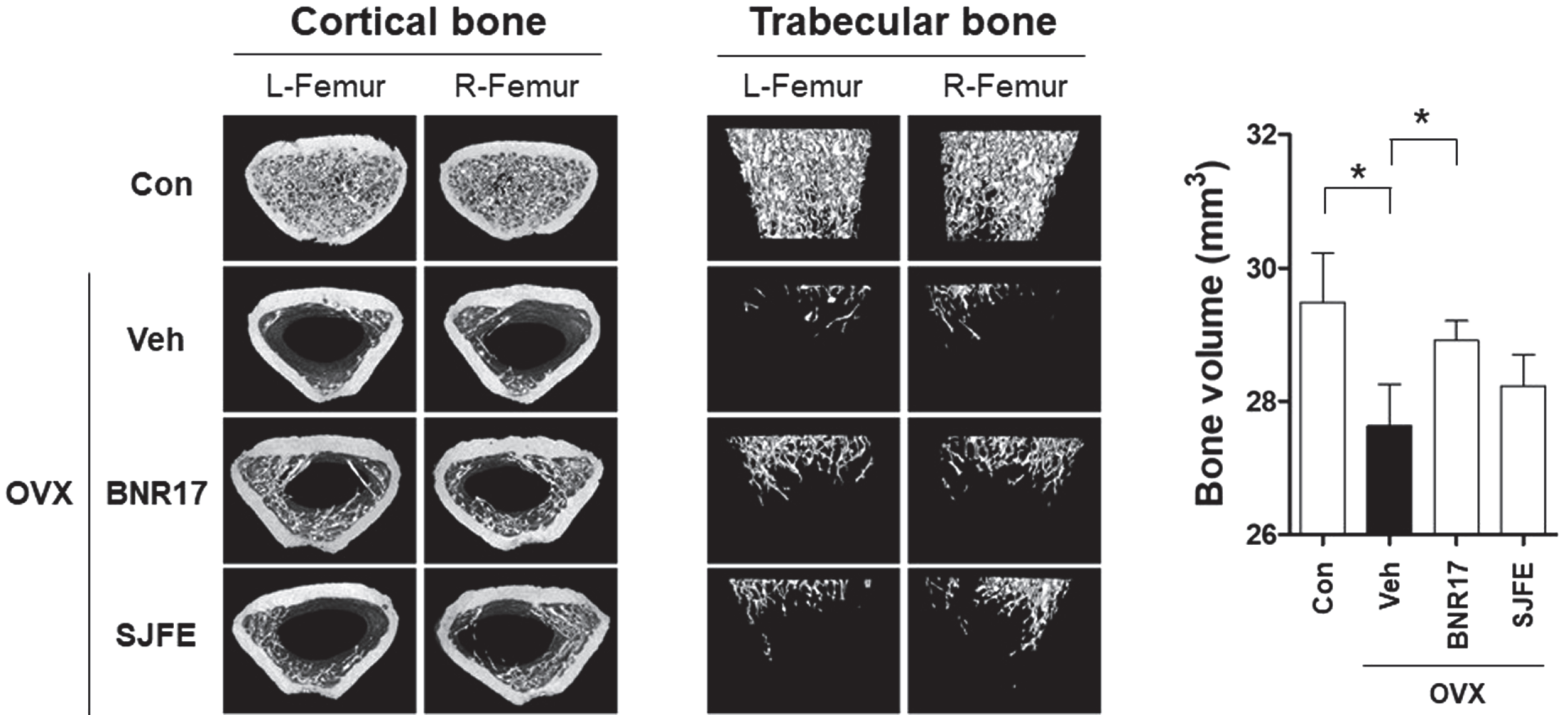
Effects of *L. gasseri* BNR17 administration on bone loss in OVX rats. Representative μ-CT images of the morphometric features of the cortical and trabecular bone (*n* = 6~8 rats per group). SJFE treatment was used to create the positive control.

Osteoporosis is a bone disease characterized by low bone density, which increases the risk of fracture [37-40]. The incidence of osteoporosis is generally higher in postmenopausal women due to low levels of estrogen. The OVX rat is a well-characterized animal model used in osteoporosis studies [41-43]; the effects of *L. gasseri* BNR17 administration on bone structure in OVX rats were examined using μ-CT. Representative μ-CT images of the distal metaphysis of the femur are shown in Fig. 4. Veh-treated rats exhibited significant bone loss compared to their control, as characterized by lower cortical bone and trabecular bone volume (Fig. 4). However, the BNR17 treated groups showed a higher bone volume than the Veh group. These results show that *L. gasseri* BNR17 administration may help prevent bone loss in OVX rats.

## Discussion

Little is known regarding the correlation between *Lactobacillus* and postmenopausal symptoms. Previous reports indicated that some strains of *Lactobacillus* species affect the process of bone formation, which is involved in calcium homeostasis [44, 45] and relieving mechanical sensitivity [46]. This may be the possible mechanism through which *L. gasseri* BNR17 has been found to improve the pain sensitivity and bone density in OVX rats.

Biochemical analysis confirmed that the administration of *L. gasseri* BNR17 led to an increase in the concentration levels of serum 5-HT-2A and calcitonin. Calcitonin, a calcium-regulating hormone, possesses potent hypocalcemic activity, which results from the suppression of osteoclastic activity. Interestingly, 5-HT-2A has been shown to affect the secretion of calcitonin by C cells in the rat thyroid [47]. Additionally, many studies have reported that calcitonin exerts analgesic effects in diverse painful conditions, including osteoporotic vertebral fractures, bone pain, and female osteoporosis [48, 49]. Therefore, calcitonin has been applied to treat postmenopausal osteoporosis, Paget’s disease, and hypercalcemia [50, 51]. Calcitonin also plays a role in the urinary excretion of deoxypyridinoline [52, 53]. In these clinical studies, calcitonin administration selectively inhibited deoxypyridinoline excretion. Therefore, we assumed from the results that calcitonin was highly effective in decreasing the bone loss. In line with these previous studies, in the present study, we demonstrated that the treatment with *L. gasseri* BNR17 significantly increased the concentration levels of both serum 5-HT-2A and calcitonin and led to a decrease in the concentration of deoxypyridinoline in OVX rats. Because we assumed that at least the administration of *L. gasseri* BNR17 could increase the serum calcium homeostasis in the host, the increase in calcitonin concentration level might relieve the postmenopausal symptoms.

Vaginal cornification analysis is commonly performed as part of in vivo assays to monitor the cellular differentiation and to evaluate the presence of vaginal epithelium cells, such as leukocytes, nucleated cells, and cornified cells [54, 55]. This result of this analysis depends on the sex steroids, particularly estrogens, as increased levels of estrogen cause vaginal cornification. Unlike previous studies, we found that *L. gasseri* BNR17 administration induced vaginal cornification, but it was not associated with an increase in the estrogen levels. It is currently difficult to explain the cause of this discrepancy. However, *L. gasseri* is one of the major microbiome species isolated from the vagina and breast milk [56-59], and several benefits for women health have also been reported [60, 61]. *Lactobacilli* adhere to the vaginal epithelial cells and help maintain the vaginal flora and homeostasis, including the cornification of the human vaginal epithelium, directly affecting the health of women [62, 63]. This implies a possible association of *L. gasseri* BNR17 with increased adhesion to the immortalized human vaginal epithelial cells (MS74), as shown in the present study (Fig. S1). The adhesion of *L. gasseri* BNR17 to MS74 cells was quantitatively analyzed using the microscopic examination, and the results indicated that *L. gasseri* BNR17 was categorized as strongly adhesive (12.2 ± 0.8 bacteria/MS74 cell). However, the ability of *L. gasseri* BNR17 to adhere to MS74 cells does not explain the mechanism of action of their menopausal relieving effect. Nevertheless, it is expected that the vaginal adhesion of *L. gasseri* BNR17 induces the changes in the vaginal microbial environment, which may help alleviate the menopausal symptoms. Therefore, further studies are required to identify the underlying mechanisms of action of such probiotic–host interactions.

The plant derived extract, *Sophora japonica* fruit extract (SJFE) is a well-known traditional Chinese medicinal herb and is of great interest owing to its diverse biological properties [22, 23, 64, 65]. According to a previous study, SJFE has been shown to alleviate some menopausal symptoms in OVX female rats, such as decreased total cholesterol, follicle-stimulating hormone, and calcium levels [23, 64]. Additionally, rexflavone, present in SJFE, has been shown to exert beneficial effects on the postmenopausal symptoms in women [66]. Although further studies are required to verify the optimal efficacy and associated mechanism of action of *L. gasseri* BNR17, its administration appears to be more efficient in increasing the serum levels of calcitonin and bone volume than that of SJFE.

In conclusion, this study suggests that *L. gasseri* BNR17 reduces the levels of bone turnover markers, thereby preventing bone loss. In addition, this study suggests that *L. gasseri* BNR17 recovered reduced vaginal cornification and hyperalgesia, which are most frequently associated with menopausal symptoms.

## Supplemental Materials

Supplementary data for this paper are available on-line only at http://jmb.or.kr.

## Figures and Tables

**Table 1:** Effect of 14 weeks treatment with *L. gasseri* BNR17 on hematological characteristics in the serum of OVX rats.

Variable	Con	OVX		

		Veh	BNR17	SJFE
ALT (U/L)	31.7 ± 9.7	51.9 ± 20.1^#^	36.2 ± 11.9*	64 ± 52.2
AST (U/L)	108.2 ± 32.2	223.9 ± 99^#^	156.1 ± 96.2	272.3 ± 153.6
BUN (mg/dL)	14.5 ± 1.6	12.6 ± 1.6^#^	12.5 ± 1.8	12.1 ± 2.8
Crea (mg/dL)	0.43 ± 0.04	0.44 ± 0	0.4 ± 0*	0.37 ± 0.1*
ALB (g/dL)	3.5 ± 0.3	3.3 ± 0.2	3.2 ± 0.1	3.4 ± 0.4

SJFE; *Sophora japonica* fruit extract, ALT; Alanine Aminotransferase, AST; Aspartate Aminotransferase, BUN; Blood Urea Nitrogen, Crea; Creatinine, ABL; Albumin.

^#^Significantly different compared to the Con group (*p* < 0.05).

*Significantly different compared to the vehicle (Veh) in the OVX group (*p* < 0.05).
