# Supplementary material for: The Effect of Lactobacillus gasseri BNR17 on Postmenopausal Symptoms in Ovariectomized Rats
Source: J Microbiol Biotechnol. 2021 Jul 19;31(9):1281–7. doi: 10.4014/jmb.2105.05032 (PMC9705893; doi:10.4014/jmb.2105.05032)
Supplement: Supplementary file 1 [file jmb-31-9-1281-supple.pdf]

J. Microbiol. Biotechnol.
https://doi.org/10.4014/jmb.2105.05032

**Fig. S1. Adhesion of *L. gasseri* BNR17 to MS74 cells. (A) Untreated MS74 cells, (B) *L. gasseri* BNR17.**

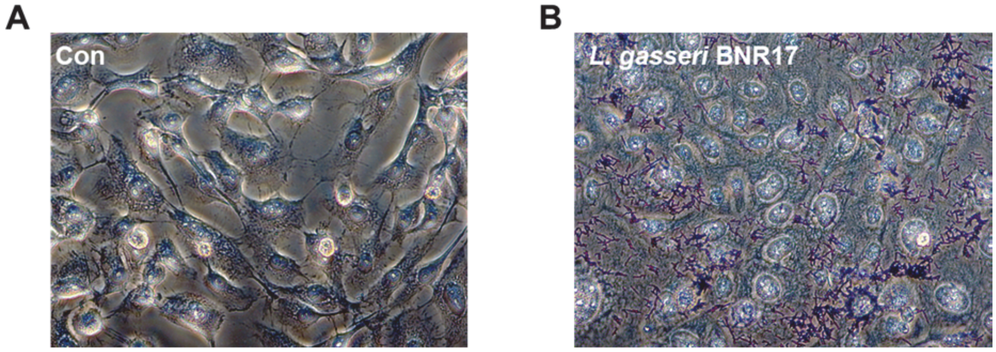


## Materials and Methods

### Adhesion assay

Immortalized human MS74 cells (kindly provided by Prof. Jae-Sook Ryu, Hanyang University College of Medicine, Seoul, Korea) were cultured at 37˚C in a 5% $CO_2$ atmosphere in DMEM (WelGene, Daegu, Korea) supplemented with 10% FBS (Hyclone, Logan, UT, USA) and 1% penicillin-streptomycin (Hyclone) in a humidified atmosphere for 2-3 days. Confluent MS74 cells were harvested and seeded in 6 well plates ($5\times10^5$ cells/well) in the presence or absence of *L. gasseri* BNR17 ($1\times10^8$ CFU/well). After 2 h of incubation, the mono-layers were washed five times with PBS, fixed with 4% paraformaldehyde, subjected to Gram staining (BD BBL, Sparks, MD, USA), and examined microscopically.
